# Anomalous Laterally Stressed Kinetically Trapped DNA Surface Conformations

**DOI:** 10.1007/s40820-021-00626-2

**Published:** 2021-05-22

**Authors:** Valery V. Prokhorov, Nikolay A. Barinov, Kirill A. Prusakov, Evgeniy V. Dubrovin, Maxim D. Frank-Kamenetskii, Dmitry V. Klinov

**Affiliations:** 1 Federal Research and Clinical Center of Physical-Chemical Medicine, Malaya Pirogovskaya, 1a, Moscow, 119435 Russian Federation; 2 A.N.Frumkin Institute of Physical Chemistry and Electrochemistry, RAS, Leninsky prospect 31, Moscow, 199071 Russian Federation; 3 Moscow Institute of Physics and Technology, Institutskiy per. 9, Dolgoprudny, 141700 Moscow, Russian Federation; 4 Lomonosov Moscow State University, Leninskie gory, 1-2, Moscow, 119991 Russian Federation; 5 Department of Biomedical Engineering, Boston University, 44 Cummington Mall, Boston, MA02215 USA

**Keywords:** DNA surface conformations, Kinetic trapping, Lateral stress, Periodically charged surface, DNA kinks

## Abstract

**Highlights:**

- DNA kinking is inevitable for the highly anisotropic 1D–1D electrostatic interaction with the one-dimensionally periodically charged surface.
- The double helical structure of the DNA kinetically trapped on positively charged monomolecular films comprising the lamellar templates is strongly laterally stressed and extremely perturbed at the nanometer scale.
- The DNA kinetic trapping is not a smooth 3D—> 2D conformational flattening but is a complex nonlinear *in-plane* mechanical response (bending, tensile and unzipping) driven by the physics beyond the scope of the applicability of the linear worm-like chain approximation.

**Abstract:**

Up to now, the DNA molecule adsorbed on a surface was believed to always preserve its native structure. This belief implies a negligible contribution of lateral surface forces during and after DNA adsorption although their impact has never been elucidated. High-resolution atomic force microscopy was used to observe that stiff DNA molecules kinetically trapped on monomolecular films comprising one-dimensional periodically charged lamellar templates as a single layer or as a sublayer are oversaturated by sharp discontinuous kinks and can also be locally melted and supercoiled. We argue that kink/anti-kink pairs are induced by an overcritical lateral bending stress (> 30 pNnm) inevitable for the highly anisotropic 1D-1D electrostatic interaction of DNA and underlying rows of positive surface charges. In addition, the unexpected kink-inducing mechanical instability in the shape of the template-directed DNA confined between the positively charged lamellar sides is observed indicating the strong impact of helicity. The previously reported anomalously low values of the persistence length of the surface-adsorbed DNA are explained by the impact of the surface-induced low-scale bending. The sites of the local melting and supercoiling are convincingly introduced as other lateral stress-induced structural DNA anomalies by establishing a link with DNA high-force mechanics. The results open up the study in the completely unexplored area of the principally anomalous kinetically trapped DNA surface conformations in which the DNA local mechanical response to the surface-induced spatially modulated lateral electrostatic stress is essentially nonlinear. The underlying rich and complex *in-plane* nonlinear physics acts at the nanoscale beyond the scope of applicability of the worm-like chain approximation.
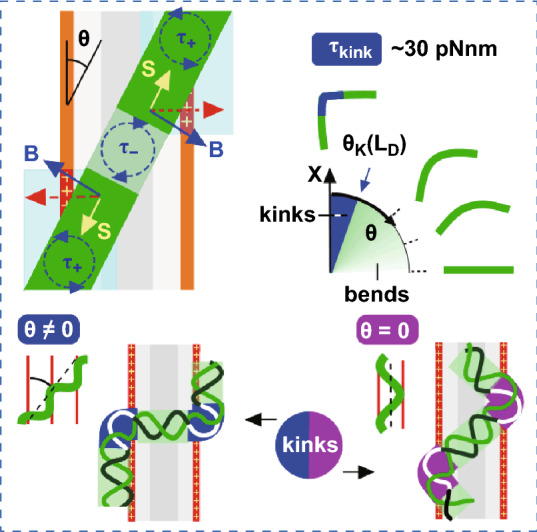

**Supplementary Information:**

The online version contains supplementary material available at 10.1007/s40820-021-00626-2.

## Introduction

Bending deformations of stiff DNA molecules in a wide range from small (thermal) to moderate (up to the nucleosomal ~ 5 nm in a radius) have long been described by the simple classical worm-like chain (WLC) model, which has become the successful theoretical paradigm for quantitatively treating a huge body of experimental data in a bulk solution in vivo and in vitro [1]. The WLC model treats DNA as a homogeneous stiff elastic rod with a quadratic bending potential and describes the linear elastic DNA behavior using a single parameter of the persistence length P ~ 50 nm. Despite almost universal applicability of the WLC model, DNA mechanical properties become essentially nonlinear under conditions of extreme mechanical bending [2–4] or stretching [5–8]. The sharp DNA bending results in the formation of discontinuous kinks, which were originally predicted by Crick and Klug some 40 years ago as localized disruptions of the regular helical structure [9]. It was shown from the experiments on cyclization of short ds-DNA fragments that a kink is induced when the DNA curvature radius reaches the threshold of about 3.5 nm [2, 10, 11]. In an alternative experimental approach, the mechanics of kinked DNA has been studied by observation of the buckling transition in the short D-shaped DNA constructs [3, 4]. The critical bending torque for kink development has been shown to be a materials parameter independent of the local base sequence and has been estimated to be about 30 pNnm [4]. Many possible candidates for kinked DNA structures have been observed in molecular dynamics simulations [12–16].

Up to now, the surface was believed to always preserve stiff native DNA structure (this is a mandatory requirement in nanotechnological applications and microscopic observations of DNA/proteins binding etc.) and is incapable of inducing any strong structural transitions in DNA. This belief implies a negligible contribution of unbalanced electrostatic forces coming from the unevenly distributed surface charges and acting *parallel* to the surface (i.e., lateral forces). Surprisingly, the validity of this assumption has never been discussed. In the microscopic analysis of surface DNA conformations observed by atomic force microscopy (AFM) for the last two decades, mainly large-scale behavior insensitive to structural peculiarities at the nanometer scale has been actively studied for DNA adsorbed on different surfaces such as mica [17–21], positively charged films on mica [21–25] and on highly oriented pyrolytic graphite (HOPG) [26–29]. Using end-to-end distance measurements, the DNA surface conformations were classified as the freely equilibrated (they are smooth at a nanometer scale and well described by the WLC model [30]) and much lesser smooth more compact kinetically trapped so-called projected DNA conformations [17]. The “projected” conformations were believed (in fact without any justification other than the large DNA bending stiffness) to follow the same WLC model as freely equilibrated conformations (corrected to fit the lower (2D) dimensionality) although many highly bent sites were observed in AFM images [22–28]. This striking contradiction with the expected WLC low-scale behavior was not recognized.

The present experimental AFM study complemented by qualitative physical analysis clearly demonstrates that DNA molecules kinetically trapped on monomolecular films comprising one-dimensional periodically charged lamellar templates experience an extremely large (overcritical) bending stress, which results in multiple kinking directly observed by AFM. In addition, the compact supercoiling anomalies and the small melting bubbles were observed and interpreted as other surface stress-induced conformational anomalies. The extreme and the spatially modulated lateral stress (bending, tensile and unzipping) makes the observed kinetically trapped DNA surface conformations principally anomalous.

## Experimental Section

### Chemicals and Sample Preparation

BamH linearized DNA plasmid pUC19 (2686 bp) and circular pBR322 (4361 bp) plasmid were purchased from Fermentas, USA. Supercoiled circular M13mp18 RF M13 DNA (7249 bp) was purchased from Takara (Tokyo, Japan). The hybrid aminated glycine-alkanes derivative (GA) NH_2_Gly_4_(CH_2_)_10_Gly_4_NH_2_ (https://patentimages.storage.googleapis.com/a9/06/b6/055897909df416/WO2007011262A3.pdf) was obtained from Nanotuning, Russia. It was early used as a surface-modifying agent allowing the deposition of ss- and ds-DNAs on the graphite substrate and there called as GM (graphite modifier) [27, 28]**.** To prepare a GA uniform film, a drop of 0.1 mg mL^−1^ GA water solution was placed on a freshly cleaved HOPG surface for a time of 10 s and then removed with a flow of nitrogen. The incomplete GA films with close-packed or isolated GA lamellae were obtained by the HOPG exposure to the diluted GA solutions with a concentration ~ 0.5 μg mL^−1^ for a time of 5–50 s. Before the deposition, DNA was dissolved in KCl or TrisHCl (pH8.0) with a salt concentration varied in the range of 0.2–20 mM. A drop of 0.5 µg mL^−1^ DNA solution was deposited on a freshly prepared GA film for a time of ~ 1 min and then withdrawn with a gentle stream of nitrogen. The DNA adsorption on freshly cleaved mica was carried out according to the standard divalent cations assisted protocol [17, 18] from a solution containing 10 mM ammonium acetate and 5 mM MgCl_2_. The mica with the adsorbed DNA was then rinsed with MilliQ water for a time of ~ 10 min and dried.

### AFM Imaging

AFM imaging in air was performed using Ntegra Prima (NT-MDT, Russia) operated in tapping mode. Ultrasharp AFM probes with a spring constant of 5–10 N m^−1^ and a resonance frequency of 150–350 kHz were used (carbon nanowhiskers with a curvature radius of several nanometers grown at tips of standard tapping mode silicon cantilevers [28]). Apart from the extreme probe curvature, the proper choice of the probe-surface interaction regime has been shown to be important [31]. The probe driving amplitude was chosen the least possible (typically ~ 10 nm) at which both tip/sample operating regimes (i.e., the attractive and the repulsive regimes [32]), occur as the operational amplitude is reduced [33]. The transition between the regimes (typically in the range of 3–5 nm) was dependent on the cantilever spring constant and the probe curvature radius. With the used ultrasharp probes, the repulsive regime provides better resolution due to the direct probe/surface contact with the reduced sample elastic deformation [31]. The AFM measurements in water were performed using a Multimode scanning probe microscope Nanoscope IIIa (Digital Instruments, USA) operated in tapping mode with commercial cantilevers NP-S1 (Veeco, USA) having a spring constant of 0.3–0.6 N m^−1^. Off-line analysis of AFM images was performed using Femtoscan software (http://www.nanoscopy.net/en/Femtoscan-V.shtm).

### Calculation of < R^2^ (L) >

The contours of DNA molecules (N > 100 for each case) were digitized using the DNA trace software [34]. Calculation of < R^2^(L) > was performed in Scilab 6 program (https://www.scilab.org/). In brief, the mean-square distance < R^2^ > in dependence on the curvilinear length L was obtained by averaging the values measured for all pairs of points separated by a length L along the chosen DNA contours with L ranged from 25 nm to the maximal traced DNA length with a 25 nm step.

### DNA Fluorescence Measurements

The measurements of fluorescently labeled DNA were conducted on Nikon® Eclipse Ti inverted epifluorescence Microscope (lens ApoTIRF × 100, NA 1.49) equipped with Andor® iXon 897 EMCCD camera and filters providing excitation for SYBR® Green at 465–495 nm and emission at 515–555 nm. DNA was stained by SYBR® Green I Sigma-Aldrich®.

## Results and Discussion

### Extensive Kinking in the Kinetically Trapped DNA Adsorbed on the Seemingly Uniform Monomolecular Films

**Fig. 1:**
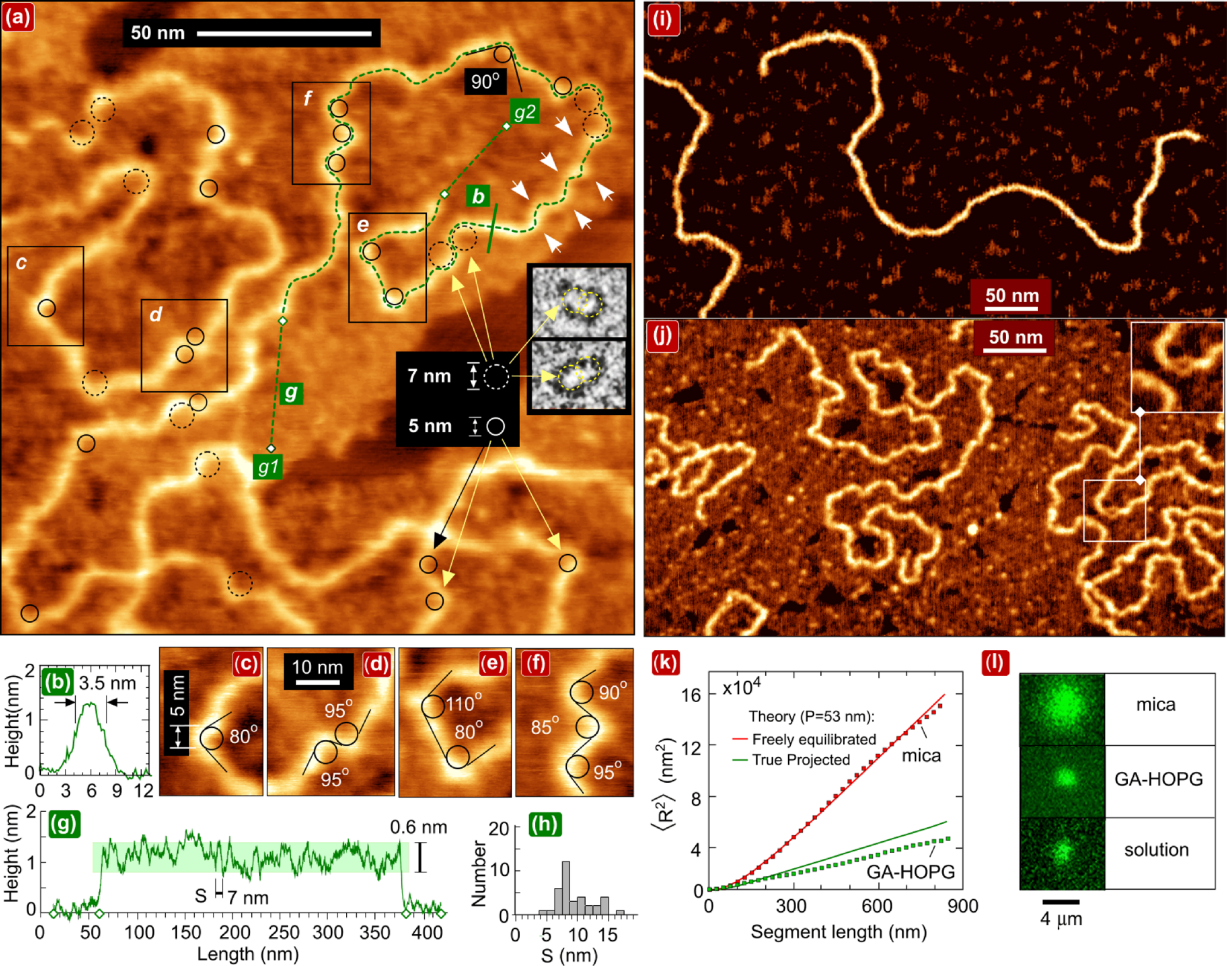
AFM imaging of kinetically trapped kinked DNA. **a** High-resolution AFM topography image in air of pUC19 DNA on the uniform GA/HOPG film. Bending undulations are shown by white arrows and kink sites are marked by black circles. The scan size is 180 nm, and the sampling resolution is 0.18 nm/pixel. The two insets on the right are EM images of kinked doubly gapped mini-circles, reproduced with the same scale from ref11 licensed under CC BY-NC 3.0. **b** The profile drawn across DNA along the green line *b* in (a). **c–f** Zooms of rectangular areas *c* to *f* in (a). **g** The longitudinal DNA profile drawn along the dashed line between points g1 and g2 in (a). **h** The histogram of the distribution of segment lengths between adjacent height minima in the longitudinal profiles. **i, j** Visual comparison of the low and large scale behavior of (i) the smooth freely equilibrated pUC19 DNA conformations on mica and (j) the undulated “projected” conformations on GA/HOPG. The inset in the top-right corner of Fig. 1j shows the melting bubble. The size of scans is 550 nm (i) and 460 nm (j). **k** Experimental dependences of mean-square end-to-end distance 〈R^2^(L)〉 as a function of the segment length for DNA adsorbed on GA/HOPG (lower branch) and on mica (upper branch). The red and green solid lines are theoretical dependences for the DNA with the persistence length P = 53 nm plotted for the 2D freely equilibrated and projected conformations, respectively, in accordance with relationships in ref 17. **l** The direct optical visualization of single fluorescently labeled lambda-DNA coils in a bulk solution and adsorbed on mica and GA-HOPG

Figure 1a shows a high-resolution AFM topography image of pUC19 DNA adsorbed on a GA/HOPG film. A striking feature in the images in Fig. 1 highly unexpected for the inherently stiff DNA molecule is the presence of multiple anomalously sharp bends, whose mutual separations are much smaller than the persistence length. Several closely spaced bends form characteristic undulations, such as those marked by white arrows in the upper right of Fig. 1a. Dashed and solid black circles with radii 3.5 nm (the critical radius of kink formation [2, 10, 11]) and 2.5 nm (an even smaller value limited by the achieved AFM resolution) are superposed in Fig. 1a, c-f on some extremely curved DNA sites where the formation of kinks is assumed. For comparison, two insets in the right represent electron microscopy images of kinked mini-circles taken from [11]. The angle between tangent vectors of adjacent DNA segments in the sites of extreme curvature is mostly in the range of 85–105° as exemplified in zooms in Fig. 1c–f, which is close to the value predicted for kinks by Crick and Klug [9]. The linear density of kinks estimated from AFM images reaches the level of several kinks per persistence length. This is about three orders in a magnitude larger than the probability to find an opened base pair for free DNA molecules in solution (~ 10^–5^ bp^−1^ or ~ 1.5 × 10^–3^ per persistence length) [35]. The longitudinal topography profile in Fig. 1g shows that the adsorbed DNA is highly and unevenly compressed. The mean-square distance 〈R^2^〉 was measured in accordance with the standard approach [17] in dependence on the curvilinear length (L) for DNA on mica (Fig. 1i) and on GA/HOPG (Fig. 1j). From the comparison of the experimental curves with the theoretical curves for the freely equilibrated and “true” projected conformations (Fig. 1k), the DNA surface conformations on GA/HOPG can be classified as “close to projected” although with a noticeable deviation. This conclusion is additionally supported by comparative *in situ* fluorescence microscopy measurements of the fluorescently labeled lambda DNA coils (Fig. 1l).

### Highly Anisotropic Orientational Impact of the Periodically Charged Epitaxial Lamellar Sublayer

**Fig. 2:**
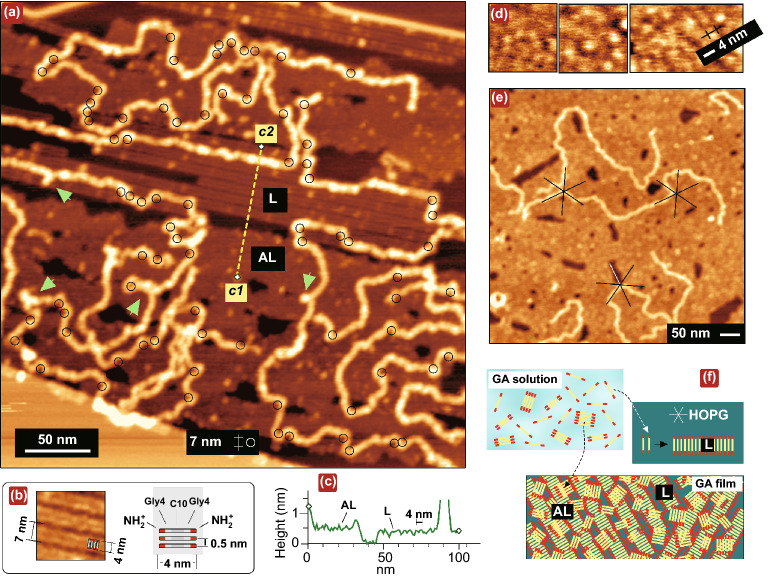
AFM imaging of the two-layer AL/L structure of GA film inducing either the projected or the template-directed DNA conformations. **a** AFM topography image in air of pUC19 DNA on the incomplete GA monolayer with morphologies of the periodic lamellar epitaxial layer (L) and the uniform granular adlayer (AL). The scan size is 345 nm. The sites of the extreme curvature are marked by the black circles with 3.5 nm radius. The Y-shaped protrusions are marked by the light green arrows. **b** The fragment of the lamellae image and the model of the molecular packing in lamellae (positively charged amino groups are shown by red). **c** The topography profile along the dashed line drawn between points c1 and c2 in (a). **d** Phase images of the granular adlayer demonstrating the grains polydispersity and a characteristic dimension.** e** AFM topography image in air showing the anisotropy of the pUC19 DNA orientation in the projected DNA conformation. Scan size 600 nm. **f** The scenario of growth of two-layer (granular adlayer/lamellar sublayer) GA film in two stages from a polydisperse GA solution

Important structural details, providing an insight into mechanism of the anomalous DNA bending, were obtained from experiments with DNA depositions on *incomplete* GA films. Such films were prepared by HOPG exposure to more diluted GA solutions. Figure 2 indicates that the seemingly uniform GA film (such as observed in Fig. 1) is in fact a two-layer system with an *in-plane* orientation of GA molecules, comprising a lower highly ordered close-packed epitaxial lamellar sublayer (L) and an upper granular adlayer (AL). DNA adsorbed *directly* on the lamellar layer adopts the “template-directed” conformation with long segments electrostatically stretched along lamellae [29]. A sketch on the right of the panel in Fig. 2b represents a model of the molecular arrangement of hybrid GA molecules in lamellae expected from the double-stripe lamellae visualization in Fig. 2a and the analysis of hexaglycylamide epitaxial structures on HOPG [31]. One-dimensional lamellae with the side-by-side molecular arrangement parallel to the crystal substrates are typical surface-grown structures formed at the first stage of adsorption of many short rod-like oligomer molecules and long (bio)polymers on various crystalline substrates such as graphite [26, 29, 31, 36, 37], mica [37–40] and silica [41]. The GA lamellar self-assembly thus renders the charge distribution in the L-sublayer one-dimensionally periodically charged.

High-resolution AFM visualization of the coarse-grained structure of GA films has been conducted (Supplementary Note S1 and Fig. 2d). It indicates that a basically similar side-by-side molecular arrangement (shown in the model in Fig. 2b) is expected for GA grains constituting the adlayer. The grains (Fig. 2d) are polydisperse oligomers with one dimension fixed and equal ~ 4 nm (i.e., GA molecular length) and the other dimension variable because of the dispersion in the aggregation number (expected in the range from 2 to about 10). The two-layer AL/L films, being more or less uniform in a topography of the upper adlayer, are highly anisotropic with respect to the local electrostatic pattern induced by the epitaxial lamellar sublayer. This anisotropy manifests itself in the frequently observed preferable orientations of the extended segments of “projected” DNA conformations along directions with sixfold rotational symmetry (Fig. 2e and Supplementary Note S2). Figure 2f shows the schematic of the formation of the two-layer architecture of GA films. The film growth proceeds in two stages. In the range of the (relatively large) used GA concentrations, the GA solution is expected to contain both the single GA molecules (major fraction) and the polydisperse GA oligomers. At the first stage, the single GA molecules rapidly self-assemble on the HOPG interface and form the highly anisotropic well-ordered epitaxial layer composed of the close-packed one-dimensional lamellae (L). At the second, slower stage, the polydisperse GA oligomers are deposited above the lamellae and form the much lesser ordered topographically featureless granular adlayer (AL).

### Model of Surface-induced DNA Kinking via the 1D–1D Delocalized Lateral Electrostatic Interaction at the Nonzero Interaxial Angle (θ ≠ 0)

The (visualized in air) DNA surface conformations, both “projected” on the compositional AL/L film (Figs. 1 and 2) and **“**template-directed” on bare lamellae (Figs. 2 and 5) are oversaturated by kinks (marked by circles in these images). The AFM *in situ* measurements in water media have also been done for the DNA adsorbed on the bare lamellae (Supplementary Note S3). They reveal multiple anomalous bending sites similar to those observed on dry samples and demonstrate that bending originates from the DNA interaction with the lamellar surface rather than from some artifacts of sample drying.

A principal assumption in the present study is that the *parallel to the surface* and normal to the DNA component of the electrostatic force is a primary and previously unnoticed source of the large bending perturbations in the DNA shape observed at the low scale. A stronger assumption (justified below) is that in the DNA adsorption on “typical” lamellar templates, the local bending stress applied to the DNA can exceed the kink formation threshold and can thus result in extreme kink anomalies. The second statement is equally applicable to the “projected” DNA adsorbed on a two-layer AL/L film with the lamellar layer buried under the featureless adlayer (such as in Figs. 1 and 2). The electrostatic force induced by the underlying lamellar sublayer freely penetrates the upper adlayer and its bending impact on the overlying DNA is therefore preserved although can be somewhat “randomized” at the low scale by the adlayer granular structure. In a similar way, the large-scale impact of the lamellar sublayer leads to preferable DNA orientations along lamellae directions visualized in Fig. 2e.

**Fig. 3:**
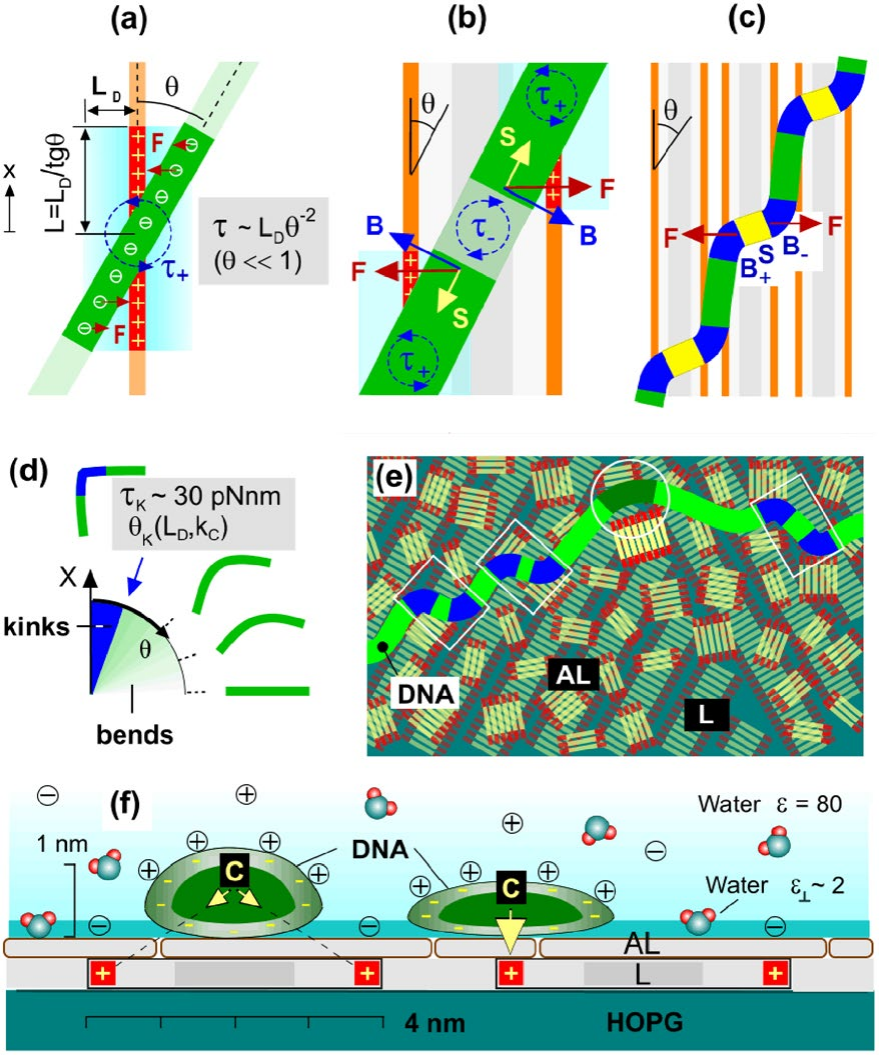
Schematics of DNA bending and kinking induced by the lateral DNA/surface electrostatic interaction (θ ≠ 0). **a** The distribution of screened electrostatic forces (red arrows) along the DNA crossing a single row of positive charges; L_D_ is the length of (salt-dependent) electrostatic screening. **b** The force and torque distribution along the DNA segment crossing the charged lamella with an inclination θ. **c** The shape of inclined DNA; the highly bent (B) and stretched (S) segments are, respectively, shown by intense blue and yellow. **d** Bends/kinks phase diagram depending on the inclination angle in the homogeneously charged rod model; Θ_K_ is the salt-dependent critical inclination for the kink formation. **e** The general model of DNA bending induced by the lateral electrostatic interaction with the underlying lamellar layer (L) and with large GA oligomers in the upper adlayer (AL); the bending units are, respectively, selected by rectangles and circle. **f** The DNA/AL/L/HOPG interfacial geometry in cross section (approximately in the scale). The ions attached to DNA and amines symbolize the counterions condensation effect reducing the DNA-lamella lateral electrostatic interaction. The electrostatic compression force (yellow arrows) depends on the intralamellar DNA position. Oriented water dipoles symbolize interfacial dielectric effect amplifying the compression force

A model of DNA undercritical and overcritical bending induced by the lateral DNA/lamellae electrostatic interaction at the inclined DNA arrangement with respect to lamellae (θ ≠ 0) is shown in Fig. 3a–c. Where the DNA intersects a single row of positive lamellar charges, the delocalized electrostatic force applied to the DNA segment induces a bending torque (τ_+_) and tends to align the DNA chain along the row (Fig. 3a), while the force distribution formed by two lamellar charge rows (Fig. 3b) induces a torque with the opposite sign in the middle (τ_-_), which leads to the appearance of a pair of bends constituting the bending unit B_+_SB_-_ with the inflection point between (Fig. 3c). As a result, a set of undulations with bent (B) and stretched (S) sites is developed as the DNA crosses several adjacent lamellae.

In this consideration, the feature of the DNA–lamella electrostatic interaction that is most important physically is its extreme angular anisotropy due to the 1D–1D interaction geometry. In the simple model of homogeneously charged intersecting rods (taken as a first approximation) [42], the bending torque τ induced by the inclined arrangement, which determines the size of strongly interacting DNA and lamellae segments (Fig. 3a), is expected to depend on the interaxial angle (θ in Fig. 3a) as:

1 $$\tau \, = \, k_C \, \left( {\pi /\varepsilon } \right) \, q_{{{\text{DNA}}}} q_{L} L_{D} \cos {\uptheta }/\sin^{2} {\uptheta }$$

where *q*_DNA_ and *q*_L_ are linear charge densities of DNA and lamellae, ε is the dielectric constant of water (ε = 80), *L*_D_ is the (salt-dependent) Debye screening length and k_**C**_ takes into account the effect of solution counterions condensation on interacting linear polyelectrolytes, i.e., on the DNA and on the side lamellar amines, highly reducing their effective charge [43–45]. The origin of the strong bending torque angular dependence at small θ angles is clearly understood. As the interaxial angle θ decreases to small values, the interaction becomes highly delocalized and effectively proceeds in the (unscreened) area highlighted by blue in Fig. 3a, b (the effectively interacting DNA segments are shown by the intense green). The interaction area length progressively increases as ~ *L*_D_/θ (at small θ). The bending torque is obtained by the integration of the product of the interaction length (*x*) and the electrostatic force (~ 1/(*x*θ)), where the lower and upper integration limits are –*L*_D_/θ and *L*_D_/θ, respectively, and x is measured from the intersection point along the positively charged row of lamellar amines (Fig. 3a). The integration provides L_D_θ^−2^ angular dependence of the bending torque. Therefore, at the sufficiently small θ, the bending torque τ reaches the overcritical threshold τ_K_ for the kink formation ~ 30 pN nm [4]. This mechanism makes the formation of a kink in the DNA segment between low-angle intersections with charged lamella inevitable *regardless of* the complex character of the polyelectrolyte interactions of the DNA with the solution counterions and with the surface. Importantly, this conclusion on the overcritical bending is also insensitive to the particular details of the DNA structure and the kink structure. For the isotropic rod model, the areas of strong overcritical bending (kinks) and undercritical bending (bends) are demarcated by the parameter of the (salt-dependent) critical tilt θ_K_, shown by the blue arrow in the phase diagram in Fig. 3d. Undercritical bends are observed for θ > θ_K_ and kinks are expected for θ < θ_K_ with a stepwise transition at θ_K_ between these two mechanical regimes (i.e., the linear and nonlinear regimes). The qualitative analysis considered above is supported by the quantitative bending torque estimates in Supplementary Note S4 that take into account the DNA helical structure directly. The DNA/lamellae configuration with a moderate interaxial tilt θ = 15° was considered there in the simpler case of DNA lying on the bare lamellar surface with no intermediate adlayer. The estimates also take the strong polyelectrolyte effects of counterions condensation highly suppressing *k*_C_ into account (*k*_C_ = 1 corresponds to the case with no effects of counterion condensation and k_C_ ~ 0.18 is obtained in case of the monovalent salt solution). The overcritical weakly salt dependent bending torques ~ 50 pNnm were obtained. Despite the roughness of these estimates, we can draw a qualitatively important conclusion with certainty: critical angle for kink formation is not vanishingly small, and kinks are therefore expected in many DNA sites inclined with respect to the underlying lamellae, i.e., at θ ≠ 0.

### Lateral Perturbative Impact in the General Case of the Coarse-grained Surface Charge Distribution

**Fig. 4:**
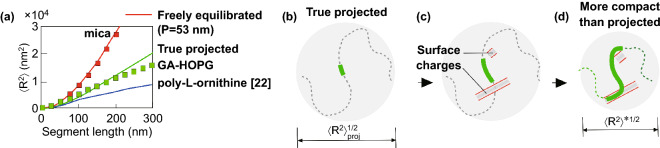
The impact of the low-scale bending on the large-scale DNA behavior. **a** Experimental dependences 〈R^2^(L)〉 of the mean-square end-to-end distance as a function of the segment length for DNA adsorbed on mica and GA/HOPG (red and green curves taken from Fig. 1k of the present study) and for poly-L-ornithine film on mica (blue curve taken from Fig. 4 of Ref. [22]). **b–d** A schematic of the bending-induced two-dimensional compactification of the DNA surface conformations below the limit of the “true projected” conformations in the course of adsorption. The DNA adsorption starts from a site shown by green in (b). Of multiple involved bending acts, only two are shown in (c). The *effective* persistence length of the conformation (c) derived from the measurements of the end-to-end distances is reduced

Up to now, the upper granular adlayer (AL) of composite AL/L films was considered only as an intermediate layer whose major function is to “randomize” the DNA anisotropic alignment by the underlying charged lamellar layer (see Supplementary Note S2). As a result, the formation of long “template-directed” DNA segments becomes inhibited but the local alignment is preserved and causes the strong local bending  considered above  in the framework of the model of the 1D-1D electrostatic interaction (Fig. 3c). In a more general interaction model, the GA oligomers constituting the adlayer also induce the additional bending in DNA. The bending appears due to the local charge misbalance randomly occurring on the DNA sides as shown schematically in Fig. 3e and in Fig. 4 below. Because of the large size of GA oligomers, the charge effectively interacting with DNA (encircled in Fig. 3e) can also be large (up to ~ 10) providing the strong local bending. Such bending resembles one induced by localized electrostatic DNA interaction with multivalent cations [46, 47] or nonspecific DNA-binding proteins where bending angles in a wide range up to ~ 100° have been reported [48]. The additional need to study the GA adlayer perturbative impact appears in the context of the observation of structural anomalies different from kinks such as the sites of the local supercoiling and melting (Sect. 3.9 below). In the proposed scenario of their formation (Fig. 7), the disturbing factor comes from the charged lamellae in the lamellar (sub)layer but the large oligomers constituting the upper adlayer have the same (i.e., lamellar) structure. The total perturbative impact of the GA/AL film becomes therefore notably more complex.

The same issues are addressed to other seemingly uniform highly positively charged monomolecular films used to facilitate the DNA adsorption on the atomically flat mica and HOPG [21–27, 29]. With the exclusion of the AFM imaging of the freely equilibrated DNA on the weakly charged mica surface [17–20], all the reported DNA images were obtained on these films and they show that the DNA adopts kinetically trapped conformations. At the kinetic trapping, the electrostatic force was believed to have only the vertical component, as the films were considered to be uniformly charged. For this reason, the lateral DNA/surface electrostatic interaction has so far been totally disregarded. The WLC model predicts that the flattened DNA adopts in this case the energetically preferable smoothly bent surface conformation without any energetically unprofitable strongly bent sites [5]. The assumption on the surface charge uniformity is, however, physically irrelevant if the distance between the surface charges is larger than the DNA diameter (or pitch). The nonzero and spatially variable lateral force component appears in this case and makes the DNA mechanically stressed and laterally perturbed. Are these perturbations weak and linear (i.e., described by the WLC model) or overcritically large and essentially nonlinear (as in the considered case of GA films) is the major issue. The so far disregarded details on the film structure (the chemical structure, dimensions and orientation of the constituent molecules, the local molecular ordering and the film in-depth architecture) become principally important. These issues need to be carefully studied in any particular case. Particularly, the extensive DNA kinking is expected on the substrates promoting the epitaxial growth of the charged one-dimensional structures (both densely and loosely packed) even if the interfacial epitaxial layer is masked by the upper poorly ordered adlayer.

### Impact of the Low-scale Bending on the DNA Large Scale End-to-end Statistics and the Persistence Length Measurements

Our nanoscale results have the immediate application to previous large-scale DNA studies providing the insight into the issue of the anomalously low estimates of the persistence length still remained open. Conformations notably more compact than “true” projected (i.e., with 〈R^2^〉 < 〈R^2^_proj_〉) have been observed for DNA adsorbed on various seemingly uniform positively charged polyamines films (polylysine and polyornitine) on mica [22]. In Fig. 4a, the experimental 〈R^2^(L)〉 dependence reported in Ref. [22] is overlaid with our data in Fig. 1k. Both data sets lie below the “true projected” line. The observed compactification was explained in terms of DNA “softening” (and the corresponding persistence length reduction) due to the partial neutralization of the DNA charge by the surface charges [22]. The surface was assumed to be uniformly charged and the DNA surface conformations were argued to be freely equilibrated (not a kinetically trapped). Our results suggest an alternative explanation: the observed anomalous compactness of DNA conformations is due to the excessive DNA bending caused by the non-uniform surface charge distribution. In accordance with general relationships [49], introducing additional bends can reduce the mean-square end-to-end distance (here in comparison with the “true projected” conformation as observed in the plot in Fig. 1k), and the calculated *effective* persistence length is correspondingly reduced. Keeping this in mind, Figs. 4b–d show the schematic of the purely geometrical explanation of the compactification of the DNA surface conformations by the impact of the surface-induced low-scale bending. It leads to the conformation more compact than “true” projected and does not need any modification of the persistence length as a physical parameter, assumed in Ref. [22], compared with the canonical value in the bulk solution. In the proposed explanation (Fig. 4b–d), the deviation from the “true projection” branch 〈R^2^_proj_〉 is induced by the excessive DNA bending which depends on fine-grained details of the surface charge distribution (see the Sect. 3.4 above). They are expected to be different for the films formed by the rod-like GA molecules in the present study and polyamines films in Ref. [22]. Direct information on the (different) structure of the poly-L-lysine functionalized mica surface is provided by the recent high-resolution AFM measurements, which resolve the individual poly-L-lysine chains loosely packed in monolayer in a non-lamellar way [25].

For the correct persistence length estimates, the surface-induced bending must therefore be taken into consideration or strongly minimized. Generally, both undercritical and overcritical bending can contribute. From this viewpoint, much more reliable estimates are expected for the freely equilibrated conformations (and not for the projected conformations) with the principally reduced surface-induced bending due to the weak DNA/surface interaction [18]. Particularly, the experimental data for mica obtained by us (upper branch in Fig. 1k) are well fitted by the canonical persistence length value  (53 nm) same as in Ref. [17]. In the so far undertaken molecular dynamics and analytical studies of the adsorption of semiflexible polymer chains based on the WLC model, the different adsorption potentials were probed, but the perturbative impact of lateral forces has not been taken into consideration as the surface was assumed to be two-dimensionally uniform [50, 51].

### Strong Uneven Electrostatic Compression of the Surface-adsorbed DNA

**Fig. 5:**
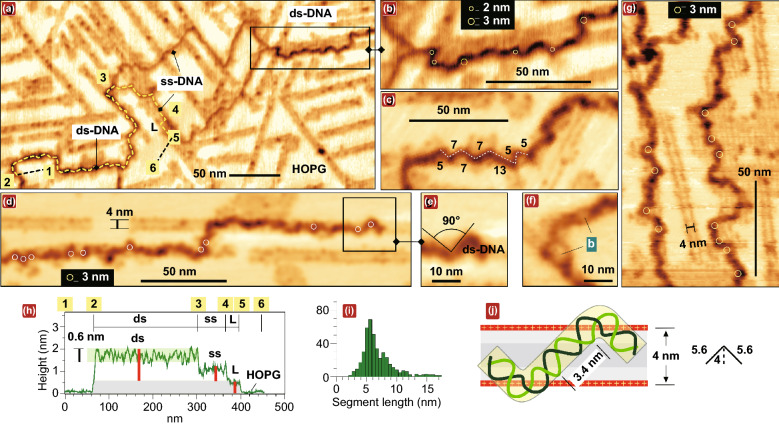
The kinking and the geometric frustrations in the shape of DNA confined between positively charged rows of lamellar amines (θ = 0) and adsorbed on lamellar domains. **a–g** Representative AFM topography images in air (inverted height palette) of supercoiled circular M13 mp18 DNA (**a, b**) and linear pUC19 DNA (**c–g**). Some extremely curved sites are marked by the overlayed circles with dimeters of 2 nm and 3 nm. The full image of DNA in (**a**) is shown in Fig. S5. **c** The geometric frustration**s** in the DNA shape manifested in the undulation segments length variation (shown in nm). **f** The small melting bubbles (*b*)**. g** The DNA geometric frustrations on the lamellar domain. **h** The longitudinal DNA profile drawn along the dashed line between points 1 and 6 in (a) that includes ss- and ds-DNA parts. **i** The histogram of the length distribution of the undulation segments. **j** The model of a segment of a minimal length terminated by the kink/anti-kink pair sticked to opposite rows of lamellar amines

Except for the extreme bending, the surface-adsorbed DNA is strongly and highly unevenly compressed. The longitudinal topography profiles show the large height variation in the range between approximately 1/3 and 2/3 of the B-form diameter. The upper and lower height levels were found to be approximately same for the projected and the template-directed DNA conformations (compare Fig. 1g and 5h below). Figure 3f shows the general schematic of the uneven compression coming from the spatially non-uniform surface charge distribution. The compression is induced by the electrostatic attraction and it is larger if the DNA is placed strictly above the underlying positive charges. This consideration is supported by the measurement of the distance between the adjacent height minima in the longitudinal profile of the projected DNA (S in Fig. 1g). The histogram in Fig. 1h shows that the adjacent depressions (expected where DNA intersects the row of lamellar amines) are separated by the characteristic distance ~ 8 nm that correlates with the periodicity of the underlying lamellar template (~ 7 nm in Fig. 2b). Importantly, the normal component of the interaction force applied from the proximal amines to bottom DNA phosphates is strongly amplified by the depressed *out-of* plane dielectric permittivity constant (ε_┴_ ~ 2) of the interfacial water [52, 53]. This water interfacial layer is 0.2–0.3 nm in thick [52, 53] and it is shown by intense blue in Fig. 3f; the strongly interacting DNA phosphates which are submerged into this layer are highlighted by dark blue circles in Fig. S4a. The inevitable consequence of such strong compression is the large broadening of the DNA width. With the additional assumption of the conserved length of the DNA strands, the broadening factor is estimated to be about 1.25 and 1.45 for the compression to 2/3 and 1/3 of the original DNA diameter, respectively, as shown schematically in Fig. 3f (approximately in the scale).

### Impact of DNA Helicity: Kink-inducing Instability in the Confined Template-directed DNA Conformations (θ = 0)

In the framework of the above consideration based on the “structure insensitive” model of the interacting homogeneously charged rods (Fig. 3a-d), generation of kink/anti-kink pairs is expected only for the inclined DNA arrangement (θ ≠ 0). Remarkably, the case of the unidirectional DNA/lamellae arrangement (θ = 0) typical for the stretched DNA segments in the “template-directed” conformations also turns out to be extremely anomalous (Fig. 5). AFM images in Fig. 5a-d show the essentially irregular non-periodic undulations at a scale ~ 10 nm. The undulations themselves and their irregular character indicate that the shape of the DNA molecule electrostatically confined between two positively charged lamellar sides becomes unstable and strongly geometrically frustrated. The lesser expressed undulations are observed in the template-directed DNA segments in Fig. 2a. The low-scale geometric frustrations are also observed in the shape of DNA lying on the lamellar domain (Fig. 5g). It is even more striking that despite a strong confinement (the separation between the positively charged amines rows is ~ 4 nm), the undulations exhibit multiple critically curved kink sites (shown by circles in Figs. 5b, d, g) in a striking difference with the Odijk behavior in nanochannels with hard walls [54].

The kink-inducing instability in the shape of ds-DNA segments is directly related with the DNA helical structure. It is not expected if DNA is modeled as a homogeneously charged rod (Fig. 3). In this case, the DNA could be placed over either the left or the right charged lamella side, whereas in Fig. 5 the DNA is placed in between and becomes kinked. The additional support for the DNA shape instability is provided by the comparative AFM observation of the extended ss-DNA segments constituting the long single-stranded loops in the torsionally stressed negatively supercoiled DNAs [23, 55, 56]. Figure 5a shows the fragment of the template-directed conformation of the supercoiled circular M13 mp18 DNA comprising such a loop (The full DNA image is shown in Fig. S5). It is observed that (having no intrinsic helicity) ss-DNA exhibits much smoother behavior than ds-DNA.

Altogether, the AFM imaging of the template-directed DNA conformations clearly indicates the action of a much more complex physics than implied by the simple model of the electrostatically interacting homogeneously charged rods in Fig. 3c. The theoretical studies in this scope have not yet been done and they are expected to be very complex**.** Only the pairwise interaction of two charged helices has been considered, and it revealed the complex angular dependence of the interaction energy with the additional energy minima different from the trivial one at ϑ = 0 [57]. A general qualitative analysis in which the DNA double helical structure is taken into account in the consideration of its interaction with the underlying periodic electrostatic relief is done in Supplementary Note S6. It specifies two expected manifestations of the DNA helicity: (1) at the DNA interaction with a *single* row of positive surface charges, the angular dependence of the interaction energy becomes more complex (Fig. S6b) and (2) when the DNA interacts with *several* charged rows it intersects, the perturbations due to the inclination-dependent lateral incommensurability of the DNA and surface periods are expected at the low scale (Fig. S6d). Despite of the lack of theory, we can propose a structural model of some characteristic building units of the template-directed kinked DNA that satisfies the strong geometrical restrictions of the observed confined DNA geometry (Fig. 5j). The typical angles in the vertices of the undulation zigzags are large (80–120°) and lie in the range of values proposed for kinks by Crick and Klug [9] (Fig. 5e), while the distribution in the length of the undulation segments has the maximum in the range of 5–6 nm (Fig. 5i). Such segments can be interpreted as structural building blocks comprising only a single DNA period terminated by the same kink/anti-kink pair as in the model in Fig. 3c but differently oriented as shown in Fig. 5j. Template-directed DNA including such building blocks exhibits the largest possible linear density of kinks ~ 1 per the DNA helical period, i.e., ~ 10 per persistence length. With the kink energy found in Ref. [4] to be equal to ~ 7 kT, the linear density of the energy saved in such DNA is estimated about two orders in a magnitude larger than in the freely relaxed DNA conformations (~ kT per persistence length).

### Non-WLC *in-plane* Physics Hidden at the Nanoscale

**Fig. 6:**
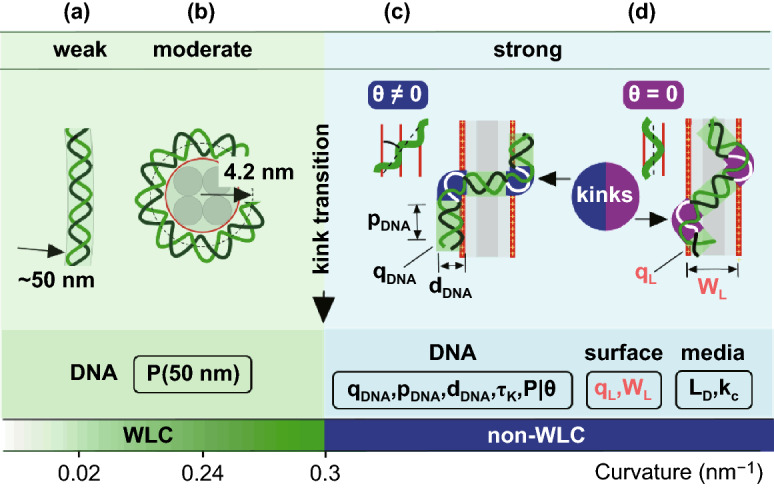
The comparison (approximately in the scale) of the undercritical weak (**a**) and moderate (**b**) DNA bending described by the WLC or similar linear elastic models and the strong nonlinear bending in the kinked non-WLC DNA conformations on a 1D periodically charged surface (**c, d**). **a** thermal fluctuations in DNA shapes. **b** DNA wrapped in nucleosomes [58]. **c, d** hypothetical models of DNA kinking at θ ≠ 0 (c) and θ = 0 (d). At the bottom, the parameters involved in the consideration and specified on the figure and in the text are listed (P is the only parameter in case of the WLC model)

Taken together, our observations in Figs. 1–5 and the above qualitative analysis open up a study in the completely unexplored area of the extreme perturbations of the DNA double helix induced by the totally overlooked impact of the lateral electrostatic stress. The underlying “*in-plane*” physics turns out an extremely complex, nonlinear and rich and has so far eluded the attention of researchers. In the particular case of the DNA interaction with the one-dimensionally periodically charged surface, it operates with the number of parameters both *external* and *internal* and the most important ones are shown in the right bottom part of Fig. 6. In contrast, the single, coarse-grained parameter of the persistence length P is used in the WLC approximation treating DNA as a homogeneous elastic rod with cylindrically symmetric cross section (left part of Fig. 6). More advanced and complex but still linear models take the DNA helicity and asymmetry into account; the consideration leads to the linear elastic coupling between bending, twisting and stretching degrees of freedom of the double helical DNA and the additional stiffness parameters with the dimensionality of length appear [8, 58].

As constituents, the physics of the lateral DNA interaction with the one-dimensionally periodically charged surface (Fig. 6c, d) includes the nonlinear DNA bending mechanics [2–4], the non-perturbative 1D and 2D polyelectrolyte electrostatic effects [43–45] and also the strong interfacial [52, 53] and the unexplored incommensurability effects. Of these constituents, the strong polyelectrolyte interaction controlled by the parameters of the Debye screening (*L*_D_) and the counterions condensation (*k*_**C**_) is the major factor driving the local DNA perturbations. The electrostatic force weakly depends on the salt concentration (via the *L*_D_ dependence). Similarly, the bending torque **τ** taken at the fixed angle θ ≠ 0 and the critical kink-inducing tilt θ_K_ are expected to weakly depend on the salt concentration; **τ** increases at the salt reduction and then saturates due to the opposite in sign contribution from the adjacent positively charged row (Supplementary Note S4). Importantly, due to the bending torque divergence at small θ (scaled as θ^−2^ in the model in Fig. 3a), there is no salt threshold for the kink formation. In accordance with this prediction, the kinks were observed on the AFM images in the whole range of salt concentrations chosen from very low to 0.2 M (although with a lesser probability at high salts). The onset of the kink-inducing instability at θ = 0 (Fig. 6d) is expected to depend crucially on the ratio of the DNA diameter *d*_DNA_ and the surface charge period W_L_. The incommensurability (W_L_/p_DNA_sinθ), i.e., the non-integer and the inclination dependent ratio of DNA and surface periods, becomes principally important at the low scale (see Supplementary Note S6). Because of the incommensurability, the perturbations along the DNA cannot be described by a periodic function. As a result, the DNA mechanical response becomes frustrated making impossible the development of the analytical models. The overcritical bending and the incommensurability are two major factors making the DNA surface conformations on the one-dimensionally periodically charged surface principally anomalous and fundamentally different from all others studied previously. Noteworthy, the incommensurability is absent as a driving factor within the WLC approximation. Interestingly, the case θ ≠ 0, which is more complex because of the angle-dependent DNA/surface incommensurability (Fig. S6d), turns out to be simpler for drawing the principally important and “structure-insensitive” qualitative conclusion that the overcritical kink-inducing bending transition at small inclinations is inevitable. Yet, the particular kink/anti-kink configuration "strictly against each other" shown in Fig. 6c should be regarded as questionable because the used in this case underlying simple model of homogeneously charged rods (Fig. 3c) completely ignores the strong impact of the DNA helicity. In contrast, the structural model of DNA confined at θ = 0 (Figs. 5j and 6d) seems closer to reality, although an obvious explanation of the kink-inducing instability of the confined double helix is lacking at present. The observed kink’s angles favor the Crick’s model of kinks with the stacking broken and hydrogen bonding saved [9]. In the strict sense, however, only the overcritical bending transition can be postulated. The DNA is additionally highly and unevenly compressed (see Sect. 3.6) and subjected to the strong unzipping stress (see Sect. 3.9 below) and, therefore, the accurate molecular modeling of the strongly bent DNA sites is necessary.

We also note that the normal to DNA *in-plane* lateral surface-induced electrostatic force was considered above as a major factor driving the extreme DNA kinking. The additional perturbative impact is expected from the recently reported specific unstacking via hydrophobic effects [59]. The hydrophobicity impact is associated in our case with the alkanes hydrophobic lamellar core (selected by the dark grey in Figs. 3f and S4). The concomitant reduction in the base-stacking energies promotes unstacking and could give rise to additional kink effects.

### Additional Perturbative Components of the Lateral Force: the Link with DNA Nanomechanics

**Fig. 7:**
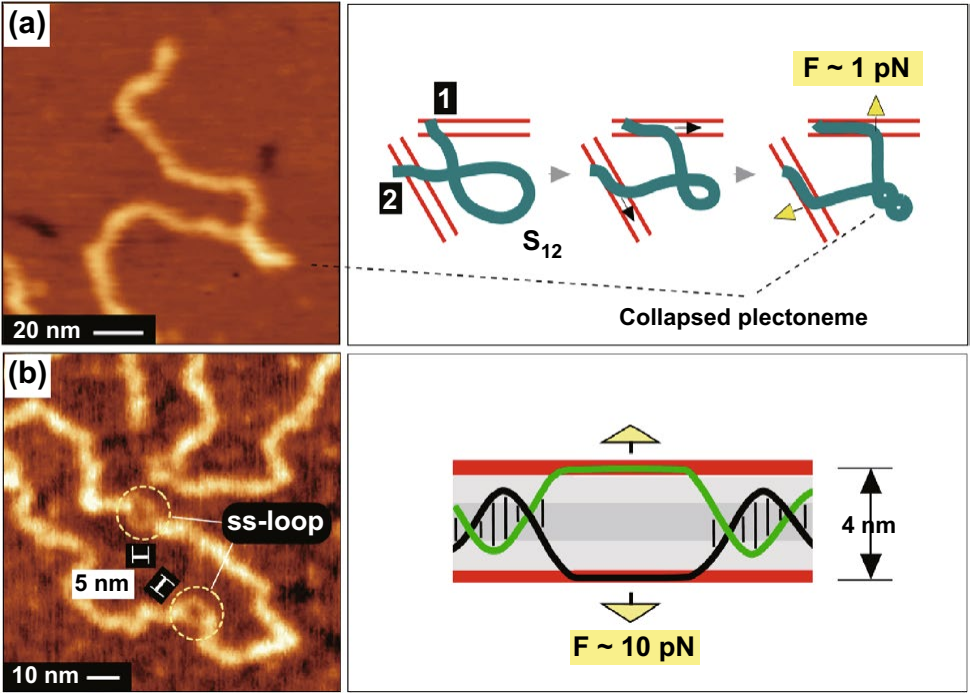
AFM images (on the left) and the probable models (on the right) of the sites of the local supercoiling and melting induced by the lateral force in the surface-adsorbed linear DNA. The positively charged rows of lamellar amines are shown by red lines. The force that controls the conformational transition is shown by yellow arrows. **a** A collapsed plectoneme model of the tight local supercoiling induced under the tensile force. **b** A model of the melting bubble stabilized by the unzipping attraction to the side lamellar amines

The mechanical response of DNA placed on the one-dimensional periodically charged surface is even more complex than discussed to this point as the double helix is subjected not only to the overcritical bending stress (the major observed effect) but also to the tangential force component, i.e., tension (S in Fig. 3b) and to the unzipping force (acting normally to the complementary DNA strands but in opposite directions). Taking the perturbative impact of these force components into account establishes an unexpected link between lateral mechanics of the surface-adsorbed DNA and nanomechanics of single DNA molecules subjected to the tensile and twisting stress in micromanipulation experiments [5–8, 60–63]. In addition to kinks, other structural anomalies such as Y-shaped protrusions (Figs. 2a and 7a) and short single-stranded loops (melting bubbles) (Figs. 1j, 5f, 7b, see also Fig. 9 in Ref. [28]) have been observed in *linear* DNAs. Such observations give an indirect indication of the presence in DNA of the strong lateral stress components different from the considered above kink-inducing bending stress. So far, the local supercoiling and melting have been reported in the literature only for the topologically constrained negatively or positively supercoiled circular DNAs [21, 23, 55, 56, 64]. Their emergence in this case is explained in a standard way by the action of the *internal factor* of the supercoiling-induced twisting stress [21, 23] (see also comments in Supplementary Note S5). Particularly, the observation of the long and short single-stranded loops is explained by the negative supercoiling induced underwinding [23]; the effect considered in many theoretical studies [65–67]. But the melting is essentially impossible in the linear DNA freely floating in a bulk solution. We therefore conclude that the observation of melting bubbles in the surface-adsorbed *linear* DNA molecules indicates the action of the surface-induced electrostatic stress as an *external* factor.

The general explanatory schematic of the formation of the sites of local supercoiling and melting is as follows. In the DNA nanomechanics, the force ~ 10 pN separates the area of the small and moderate DNA mechanical perturbations in a linear elastic regime and the strongly nonlinear elastic effects with the large rearrangement in the double helical structure [8]. On the other hand, the force-distance plot in Fig. S4c shows that the lower force in the range of several pN is easily reached, as it can be applied even to a single DNA phosphate close to the row of lamellar amines. Such tension applied *along* the DNA induces the local plectonemic transition to the tightly supercoiled “collapsed plectoneme” state if the DNA is additionally torsionally stressed [7]. The “collapsed plectoneme” model is therefore the most suitable candidate to describe both the topology and the compact structure of the Y-shaped protrusions. Figure 7a shows the probable scenario of the “collapsed plectoneme” formation in the course of the DNA adsorption. The shown by the yellow arrows permanent tension F (and not the periodical tension induced by the interaction with the underlying lamellar template) can be caused by the DNA spreading along the non-collinear lamellae starting after the DNA fixation in two sites (marked as “1” and “2”) separated by the curvilinear length S_12_. The necessary level of the under- or over-winding (the second prerequisite for the plectoneme formation [7]) can be reached by two ways. It can be inherited from the topology of the original DNA 3D-conformation in the bulk solution (such as the loop in Fig. 7a). The additional contribution comes from DNA twist deviation (Δθ_12_) from the equilibrium value due to thermal fluctuations in the bulk solution. In accordance with [8], 〈Δθ_12_^2^〉 = S_12_/C, where C ~ 100 nm is the low-force torsional persistence length. The twist fluctuation magnitude is therefore large for the large length S_12_ and the DNA becomes under- or overwound at the adsorption. The general model of the melting bubble is shown in Fig. 7b. It implies the action of the larger force ~ 10 pN, which seems to be reached by summing over several adjacent DNA phosphates. In the single-DNA micromanipulations, the complementary DNA strands can be mechanically separated by applying a force ~ 10 pN normal to the strands and in the opposite directions to force them apart [8, 60–62]. The unzipping geometry is reproduced, if the DNA is placed between the positively charged lamella sides and the force applied to single strands is sufficient to induce their separation. The separated single strands then form a stable melting bubble by sticking to the opposite lamella sides such as shown in Fig. 7b. In melting bubbles, the separation between the single strands was typically 4–5 nm (as shown in Fig. 7b) in accordance with the expectations of the model. The general sketches in Fig. 7 need the specification in details but they clearly demonstrate that the introduction of the lateral force makes the full DNA *in-plane* mechanical response extremely complex. Furthermore, both the linear and nonlinear response is expected because of the intrinsically large strength of the lateral DNA/surface electrostatic interaction. The issue is completely open for study.

### Future Experimental Perspectives

We finally note that introduced prominent DNA structural anomalies can be detected by the routine high-resolution AFM as shown in the present study. The detailed information on the pitch, bend and twist perturbations is, however, hidden at the sub-nanometer scale. In order to acquire this information, the extreme microscopic resolution allowing the visualization of both strands of the DNA double helix is necessary. Such a capability is demonstrated by the modern FM-AFM measurements, which have been done up to now only for DNA adsorbed on a mica substrate [68–72]. Interestingly, even in this case (of the freely equilibrated conformations and a much weaker DNA/surface interaction), the large deviations from canonical B-form dimensions have been observed [68, 72].

Another important point needs to be especially stressed in the context of the overlap with the high-force DNA mechanics. In contrast with the uniform external stress applied to DNA in micromanipulations, the surface force is spatially modulated. The elastic energy must therefore also include the large strain gradients terms [8] (both lateral and normal to the surface). Keeping also the coupling between bending, twisting and stretching degrees of freedom of the helical DNA in mind [8, 58] as well as the complex general force-torque DNA phase diagram [7], the new highly perturbed conformational states are highly expected to emerge in the surface-adsorbed DNA. The AFM visualization with a sub-helix resolution would make it possible unambiguous elucidating of all such conformational states. These intriguing issues are addressed for future studies.

Potentially, the periodically charged lamellar templates can be used as active “nanostages” to locally probe the linear and nonlinear DNA mechanical response. In this way, the interfacial electrostatic force field (both lateral and normal to the surface), i.e., the local stress, can be reconstructed from the visualization of charged lamellae sides. Complementary to this reconstruction, the local strain (i.e., perturbations in the pitch, bend and twist) can be derived from the visualization of the DNA double helix. The stress–strain dependence can be locally probed in such a way in any selected DNA molecule.

## Concluding Remarks

The present experimental study complemented by qualitative physical analysis clearly demonstrates for the first time that DNA molecules adsorbed on a surface can be extremely mechanically laterally stressed. The physical reason of the presence of the strong stress is the *in-plane* unbalanced electrostatic forces coming from the coarse-grained surface charge inhomogeneity completely disregarded so far*.* It was strongly argued that the case of the one-dimensionally periodically charged surface is exclusive. We show that kink anomalies stem from the anisotropy of the 1D/1D electrostatic interaction (i.e., DNA with underlying linear rows of surface charges it crosses with the inclination): the bending torque diverges at small DNA/row inclination angles and therefore inevitably reaches the overcritical level where the mechanical DNA response locally changes from the linear elastic to nonlinear kink-inducing. The kinking inevitability at small interaxial angles via the 1D-1D electrostatic interaction is the most important conclusion of the present study. This conclusion is “structure insensitive” and can therefore be equally applied to other semiflexible polyelectrolytes adsorbing on a charged surface. In addition, the striking kink-inducing mechanical instability was observed in DNA confined between the positively charged lamella sides. The linear density of kinks reaches in this case the highest possible value (~ 1 kink per the DNA helical period) making the continuity of the DNA helical structure completely broken. Except for kinks, the anomalous sites of the local supercoiling and melting have been observed. They were interpreted as other lateral stress-induced conformational anomalies by invoking the DNA nanomechanics. Low-scale bending naturally explains the anomalously low values of the persistence length based on the previous large-scale studies of the kinetically trapped DNA conformations. But even if the WLC model provides more or less satisfactory predictions on the two-dimensional dimensions of the surface-adsorbed DNA at the large scale, it can be strongly failed at the nanometer scale.

The lateral stress has not been considered so far as a critically important factor driving the DNA adsorption and determining the DNA conformational states. Many former conjectures on the surface-adsorbed kinetically trapped DNA need therefore to be revised or rejected as oversimplified. In the general case, the DNA behavior during the adsorption on the surface is not just a simple smooth 3D—> 2D conformational flattening (so far assumed for the whole wide class of the kinetically trapped projected DNA surface conformations), but is a complex nonlinear *in-plane* mechanical response on the strong and spatially variable lateral electrostatic force. Under the action of the strong lateral electrostatics, the DNA double-helix behaves not as a semiflexible polymer, as occurs in the bulk solution, but in a quite opposite way as a “fragile” linear structure. Being adsorbed, DNA forms at a nanoscale a principally anomalous segmental conformation of a strongly geometrically frustrated one-dimensional system which is oversaturated by multiple discontinuous kinks and can also be locally supercoiled and melted*.* The practically important result is that the surface generally cannot be considered as a passive agent leaving native DNA B-form intact. In a wide sense, because of the spatial modulation of the lateral stress and its overcritical value, such DNA conformations are fundamentally different from all others studied previously. The underlying rich and complex DNA/surface interaction physics and the nonlinear *in-plane* DNA mechanics were eluded the attention of researches so far. The paradigm shift from the assumed so far weakly perturbed DNA surface conformations described by the canonical worm-like chain approximation to the highly perturbed DNA surface conformations is dramatic. The obtained results uncover the extreme structural complexity arising at the nanometer scale in such strongly laterally stressed and principally anomalous DNA and emphasize the need to develop relevant theoretical and experimental approaches for their study.

## Supplementary Information

Below is the link to the electronic supplementary material.

Supplementary file1 (PDF 692 kb)
