# Supplementary material for: Anomalous Laterally Stressed Kinetically Trapped DNA Surface Conformations
Source: Nanomicro Lett. 2021 May 22;13:130. doi: 10.1007/s40820-021-00626-2 (PMC8141082; doi:10.1007/s40820-021-00626-2)
Supplement: Supplementary file 1 — Supplementary file1 (PDF 692 kb) [file 40820_2021_626_MOESM1_ESM.pdf]

Supporting Information for

Anomalous Laterally Stressed Kinetically Trapped DNA Surface Conformations

Valery V. Prokhorov[1, 2,] *, Nikolay A. Barinov[1], Kirill A. Prusakov[1, 3], Evgeniy V. Dubrovin [1, 4], Maxim D. Frank-Kamenetskii[5,] *, Dmitry V. Klinov[1, 3,] *

[1]Federal Research and Clinical Center of Physical-Chemical Medicine, Malaya Pirogovskaya, 1a, Moscow 119435 Russian Federation

[2]A.N.Frumkin Institute of Physical Chemistry and Electrochemistry, RAS, Leninsky prospect 31, Moscow, 199071, Russian Federation

[3]Moscow Institute of Physics and Technology, Institutskiy per. 9, Dolgoprudny, 141700, Moscow, Russian Federation

[4]Lomonosov Moscow State University, Leninskie gory, 1-2, Moscow 119991, Russian Federation

[5]Department of Biomedical Engineering, Boston University, 44 Cummington Mall, Boston, MA02215, USA

*Corresponding authors. E-mail: vprokh@phyche.ac.ru (Valery V. Prokhorov); mfk@bu.edu (Maxim D. Frank-Kamenetskii); dmitry.klinov@niifhm.ru (Dmitry V. Klinov)

Supplementary Note 1

High-resolution AFM imaging of the coarse-grained structure of the top adlayer of the two-layer interfacial monomolecular GA film and its origin

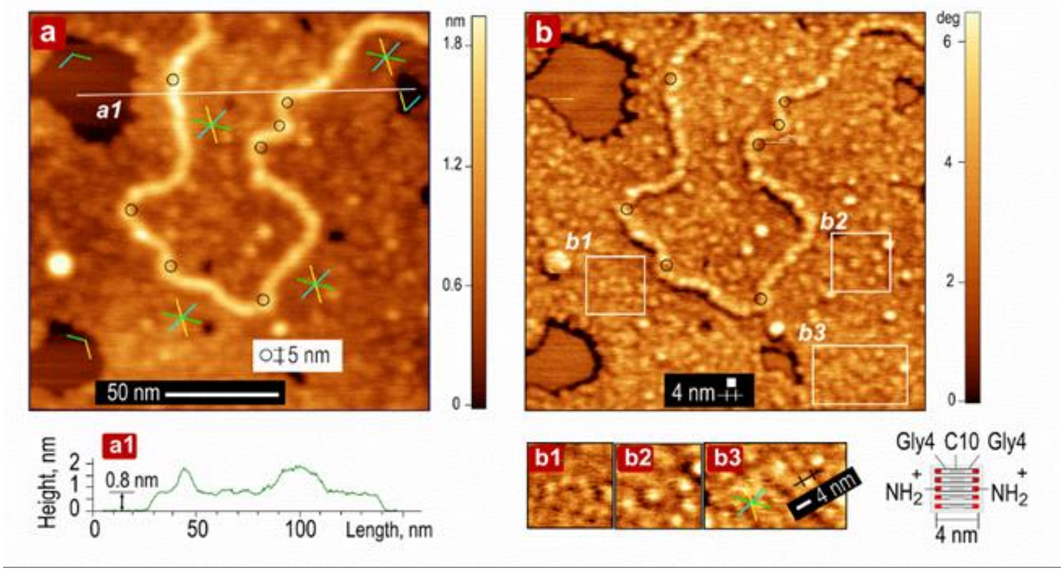

Fig. S1 High-resolution AFM imaging in air of the coarse-grained structure of GA film and its architecture (**a, b**). The (a) height and (b) phase images of granular GA film with adsorbed pUC19 DNA. The film preparation conditions: c(GA)=10 μg/ml, t=20 sec. The scan size is 170 nm. Oriented in the same way asterisks and 120° angles show preferable orientations of extended DNA segments and sides of holes in the GA film respectively. The superposed circles 5 nm in diameter mark the DNA sites with extreme curvature. (a1) The height profile along the white line *a1* in (a). (b1-b3) zooms of areas *b1-b3* in the phase image (b) demonstrating the grains polydispersity and their characteristic dimension.

Figure S1 shows the typical high-resolution image in the topography (a) and phase (b) channels of the coarse-grained structure of the compositional GA AL/L film with a fragment of the DNA molecule. The polydisperse grains have variable dimensions around 4-5 nm. The height profile in the panel (a1) shows the low thickness of the film equal to the double monolayer height (estimated to be ~0.4 nm [S1]. This indicates that GA molecules in grains are close-packed in a single layer similar to those in the lamellar layer as shown in the model in the right bottom of Fig. S1b. The side-by-side arrangement is promoted by the hydrogen-bonding in β-sheets formed by Gly4 segments [S1]. The grains are therefore flat GA oligomers with one dimension fixed and equal 4 nm (i.e. GA molecular length) and the other dimension variable because of the dispersion in the aggregation number. It is expected in the range from 2 (GA dimers 4 nm *1 nm in size) to about 10 (GA oligomers with the width 4 nm and the length 0.5 nm *10 = 5 nm). The phase images b1-b3 are duplicated in Fig. 2d of the main paper. In Fig. 2a, both morphologies, i.e. lamellar (L) and the granular (AL/L) are observed.

Supplementary Note 2

The anisotropy of the DNA orientation in the projected and template-directed surface conformations

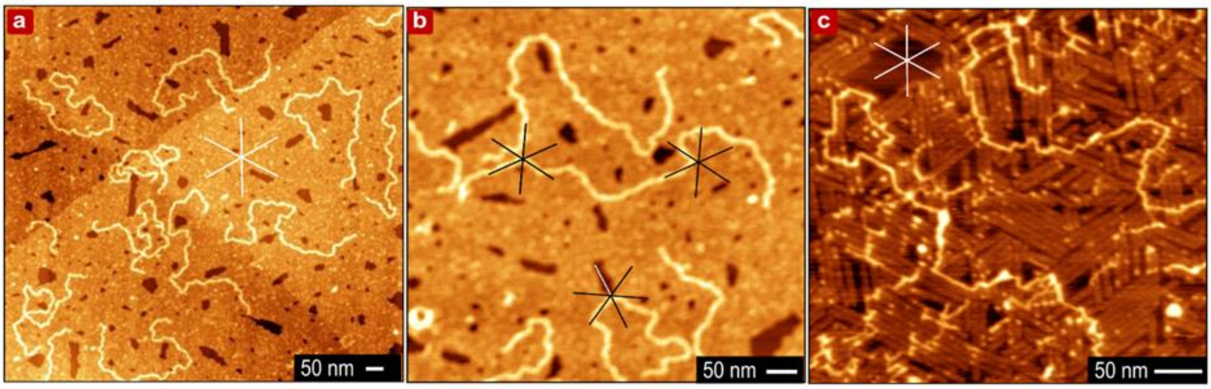

Fig. S2 The anisotropy of the DNA orientation in the projected and template directed surface conformations. (**a,b**) The large-scale alignment along preferable directions with the six-fold rotational symmetry of pUC19 DNA on the topographically uniform GA film. (**c**) The segmented template-directed pUC19 DNA conformation on bare GA lamellae. The film preparation conditions: c(GA)=10 μg/ml, t=20 sec (a,b) and c=0.5 μg/ml, t=20 sec (c). AFM imaging was conducted in air. Scan size: a) 1000 nm, b) 600 nm, c) 400 nm.

The strongly anisotropic electrostatic impact of the epitaxial lamellar monolayer buried under the top granular adlayer is manifested in the notable angular anisotropy of the frequently observed long DNA segments. For the samples with the linear holes in the GA film (which are assumed to point out at the directions of the lamellar growth), the long DNA segments are mostly parallel to the holes' directions (Fig. S2a and b). Similarly, in Fig. S1a, the preferable DNA orientations along directions with the six-fold rotational symmetry are shown by the overlayed coloured asterisks. Figure S2b is inserted in Fig. 2 of the main paper (designated there as Fig. 2e).

Figure S2c shows the topography image of DNA adsorbed on a bare lamellar template with the strong fixation of the DNA orientation along the lamellae. No qualitative differences between the projected (Fig. S2b) and the template-directed (Fig. S2c) DNA conformations is observed; they are distinguished only by the angular anisotropy degree.

Supplementary Note 3

The AFM imaging of the anomalous DNA bending in water media

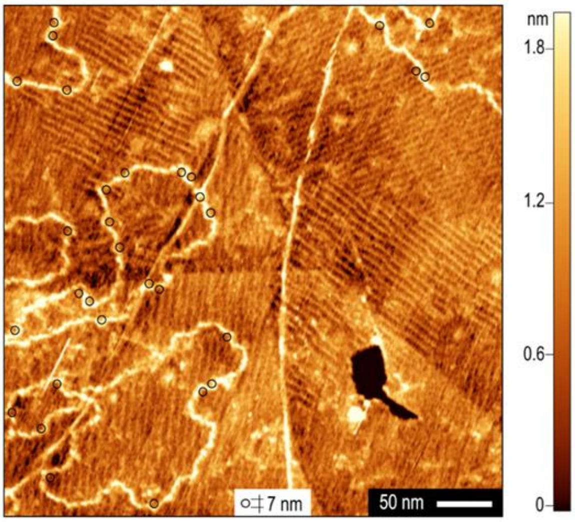

Fig. S3 The in-situ AFM topography imaging in water media of pBR322 plasmid DNA adsorbed on GA lamellar domains. The DNA adsorption was from 0.2 mM Tris-HCl buffer (pH7.4). Scan size 440 nm. Anomalously curved DNA sites are marked by superimposed circles with a curvature radius of 3.5 nm.

The AFM *in-situ* measurements in water media (Fig. S3) show that DNA chains adsorbed on bare lamellae have multiple anomalous bending sites similar to those observed on dry samples. We can conclude that the essentially irreversible nonlinear reorganization in the DNA duplex (i.e., kink formation) originates from DNA interaction with charged one-dimensional lamellar domains in the water medium rather than from some artifacts of sample drying. In the case of small linear elastic DNA deformations in water, they would reversibly relax in a dry sample as soon as the electrostatics stress disappears (for the DNA in air).

Supplementary Note 4

The estimation of the bending torque induced by the lateral DNA/lamella electrostatic interaction

Figure S4b shows the DNA/lamella cross-sectional geometry used in bending torque estimates for the particular configuration with a moderate interaxial tilt $\theta = 15^{o}$ shown in Fig. S4a. The DNA lateral size was chosen equal to the B-form diameter (2 nm), and the vertical dimension was compressed to 2/3 of the B-form diameter (the upper bound of AFM observations). The major contribution to the DNA bending is provided by two side rows of lamellar amines A_1 and A_2 (selected by red); the additive contribution of the adjacent lamellae is weak. The cross-section indicates the systematically larger contribution to the DNA bending of bottom phosphates (black) compared with top phosphates (white).

We note that both the DNA and linear rows of lamellar amines are highly charged polyelectrolites: their reduced dimensionless linear charge density (q'_{DNA} and q'_{A}) is larger than unity, $q'_{DNA}= l_{DNA}/l_B= 3.9$ and $q'_A= l_A/l_B= 1.4$, where l_{DNA}=0.17 nm and l_A=0.5 nm are the respective axial charge spacings in DNA and in lamellae and $l_B=e^2/\varepsilon_w kT$=0.7 nm is the Bjerrum length in water (ε_w=80). Therefore, the DNA/lamella electrostatic interaction includes the large contribution of short-range effect of counterion accumulation from the surrounding ion atmosphere on the linear polyelectrolites, which highly reduces the effective charge of polyelectrolites to the value $q''_{DNA} = q''_A$ =1 [S2, S3, S4]. Very roughly, this means that the interaction of DNA phosphates with amines is reduced at a short range $r<L_D$, and a long range $r>L_D$ (L_D is the Debye screening length) by a multiplicative factor $q'_{DNA}q'_A \approx 5.5$ (i.e. the coefficient k_C in formula (1) of the main paper equals 1/5.5=0.18). For comparison, Fig. S4c shows both the exactly calculated force-distance curve ($F \sim 1/\varepsilon_d r$) for the

interaction of a single DNA phosphate with a row of amines in the purely dielectric media with ε_d=80 (i.e., as for water but with the absence of short range counterion condensation effects) and that expected for the monovalent salt water solution in the low salt limit (blue curve, not relevant at very small distances r<<L_D [S5]).

The total bending torque can be obtained by summing over half of all DNA phosphates between DNA/lamella intersection points selected by the large blue rectangle in Fig. S4a: $\mathbf{M = \sum x_i (F^1_{yi} + F^2_{yi})}$

where x_i and $F^{1,2}_{yi}$ and are the respective arms and y-projections of the forces $F^{1,2}$ applied to i-th phosphate from opposite rows of lamellar amines A_1 and A_2 respectively. For simplicity of calculations, we used the DNA helical period of 10 b.p. and the simplified model expression 2pN exp(-r/L_D)/r for the interaction force (r is expressed in nm), which includes the exponential reduction of the interaction due to the Debye screening at large distances (r>L_D), the strong 1/r force dependence at small distances and the strong effect of counterion condensation (the prefactor 2pN). The DNA was assumed adsorbed on a bare lamella; the expected random (and strong) electrostatic perturbation from the adlayer of charged GA oligomers has been disregarded because it is currently difficult to estimate. With these simplifications, the torque values seem to be underestimated for two reasons. First, because they ignore the strong increase in the interaction force magnitude at a very small distance (r<<L_D) [S5]. Second, at the observed strong DNA compression its width becomes notably larger than shown Fig. S4 a, b (assuming only the vertical compression with no width increase). The compression-induced width increase makes the DNA phosphates closer to the lamellar amines and the lateral interaction force increases.

The bending torque estimates with Debye screening length (L_D) taken for four ionic strength values (I) are shown in Table 1. The bending torque expectedly increases as the electrostatic screening is reduced at smaller ionic strength. The bending torque saturation at $L_D \geq$ 2 nm (I≤20 mM) is explained by the opposite in sign contribution from the adjacent charge row (A_2, bottom in Fig. S4a) which increases as the screening length increases. The saturation is expected when two unscreened area (highlighted by blue background in Fig. 3b of the main paper) meet each other in the lamellae middle, i.e. at the Debye length equal the half of interlamellar period (~2.5-3.5 nm keeping the gaps between lamellae in mind). This condition is reached at the ionic strength of about 7-20 mM.

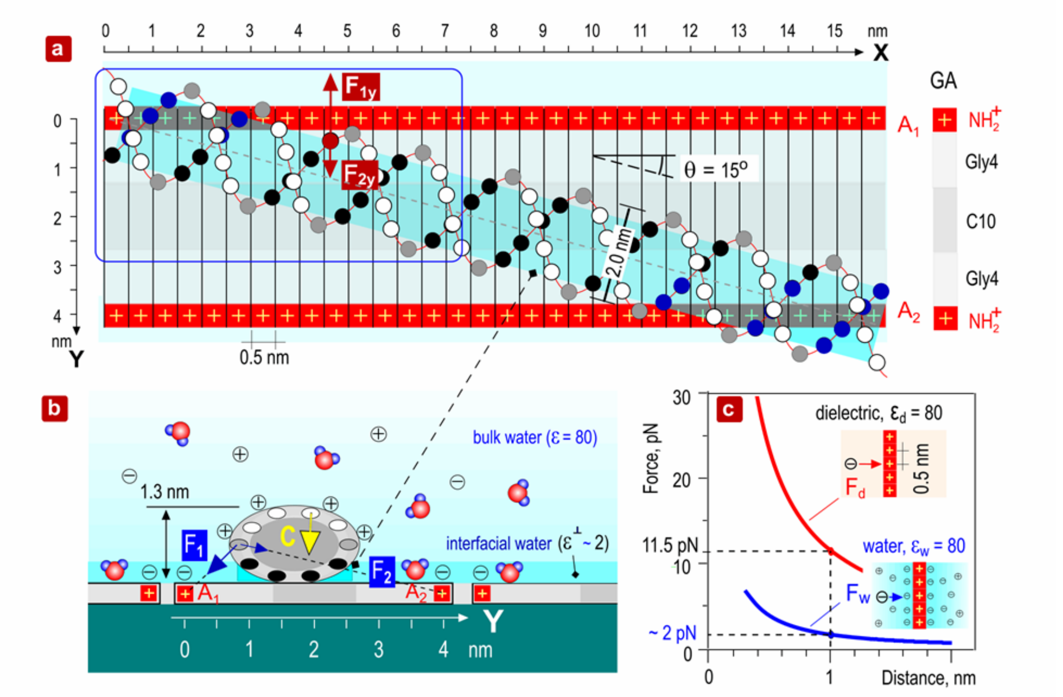

Fig. S4 A schematic of the DNA/lamella electrostsatic interaction. (**a**) The DNA/lamellae configuration (top view) with moderate interaxial tilt $\theta = 15^o$ for bending torque estimates. The DNA period is chosen to be 10 b.p. for simplicity. The top and bottom DNA phosphates are respectively shown by white and black circles; the positively charged rows of lamellar amines A_1 and A_2 are shown by red. The proximal to the lamellar amines bottom DNA phosphates which are submerged into the interfacial water layer are additionally highlighted by dark blue circles. (**b**) The DNA/lamella interfacial geometry and forces in the cross section normal to the lamella. Negative ions against lamellar amines and positive ions on the DNA symbolize the counterions condensation. The deep blue highlights the interfacial water layer with the highly suppressed out-of-plane dielectric constant $\varepsilon^{\perp}$<<80. (**c**) The force-distance curves for the interaction of a single DNA phosphate with a row of amines in a pure dielectric with ε_d=80 (red curve, exactly calculated) and in the water media in the low salt limit expected after taking the counterions condensation effect into account (blue curve).

Table 1 Estimations of the bending torque induced by the DNA/lamella electrostatic interaction at different Debye screening length (L_D) for the DNA tilt θ=15°

L_D, nm	1	2	4	10
I, mM	90	22.5	5.6	0.9
Torque, pNnm	37.1	52.6	56.1	57.6

The salt dependence of the critical kink-inducing tilt θ_K is expected to be weak. In the 1D-1D interaction model (Fig. 3a) θ_K is determined from the relationship (1): $\tau_K = k_C\ (\pi/\varepsilon)\ q_{DNA}\ q_L\ L_D \cos\theta_K /\sin^2\theta_K$, where τ_K=30 pN nm. With the additional assumption that θ_K is small, it is expected that $\tau_K \sim L_D/\ \theta^2$ and, therefore, $\theta_K \sim (L_D/\ \tau_K)^{1/2} \sim \tau_K^{-1/2}\ c^{-1/4}$.

For comparison with our estimations, recent all-atom MD simulations of the interaction of two closely spaced 20 b.p. DNA helical oligomers placed in the solution with the ionic concentration of 0.15 M can be considered which take into account the helical DNA structure implicitly [S6]. In the reported tilt range of 10-30°, they give the estimate of the bending torque, which is large and comparable with the threshold for the kink transition: $\tau \sim dE/d\theta \approx 9kT/rad = 36$ pNnm.

Supplementary Note 5

The AFM imaging of the template-directed conformation of the negatively-supercoiled circular DNA

Figure S5 shows the template-directed conformation of the negatively-supercoiled circular M13 mp18 DNA. The striking feature is the long single-stranded loop. The DNA melting temperature is estimated to be notably higher than the deposition temperature (T_m(0.2mM) ≈ 40-50 ^{o}C [S7]). Similar long loops have been observed by the electron microscopy for the supercoiled circular replicative form DNA (RF I) of coliphage M13 [S8] and by AFM for shorter supercoiled plasmids [S9, S10]. The emergence of the long loops and their length were correlated with the degree of superhelicity [S10]. These features were explained in ref. S10 by the DNA underwinding induced by the negative superhelicity; the effect considered in many theoretical studies [S11, S12, S13]. The loops appear at the expense of partial twist release; they absorb part of the undertwisting in the negatively supercoiled DNA. The M13 mp18 molecular length is ~2500 nm (7249 bp), and the average contour length of the single strands observed in AFM images was about 200 nm corresponding to ~94 nm of the ds-DNA, i.e. it is equivalent to the negative superhelical density ~ -0.04.

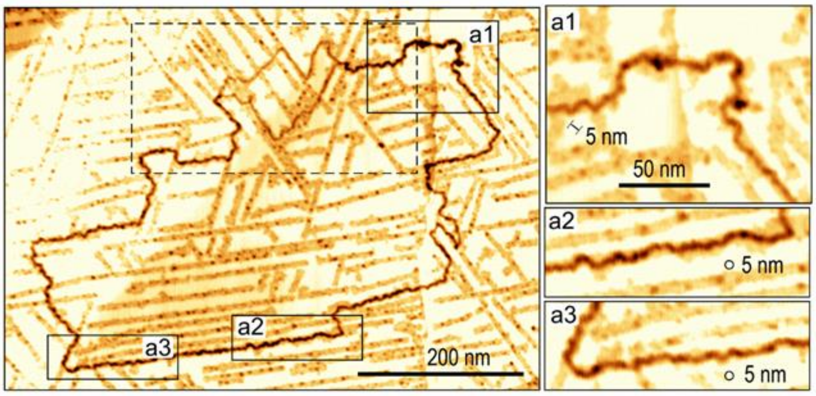

Fig. S5 AFM topography image in air (inverted height palette) of the template-directed conformation of the supercoiled circular M13 mp18 DNA on the loosely packed GA lamellae. The deposition conditions: 0.2 mM Tris-HCl, pH8.0. The magnified view of the dashed rectangle is shown in Fig. 5a of the main paper. The zooms (a1)-(a3) to the right demonstrate the kink-inducing instability of ds-DNA segments confined between positively charged lamellar sides.

Supplementary Note 6

The effects of the DNA helical structure on the interaction with the one-dimensionally periodically charged surface: a qualitative consideration

The electrostatic interaction of helical DNA with the one-dimensionally periodically charged surface hasn't been theoretically considered so far and its general analysis is expected to be very complex. Simplifying, the DNA helical structure is expected to be manifested in two ways:

First, the angular dependence of the interaction energy of the DNA double helix with a *single* row of positive surface charges (Fig. S6a) becomes not trivial (Fig. S6b). Besides of deep narrow minimum at $\theta=0$ (the single present in the model of homogeneously charged rods in Fig. 3a of the main paper), it contains two local competing minima corresponding to the contributions from the energetically preferable configurations at angles $\approx\pm62^{o}$ (=atan($p/\pi D$). In these configurations, either the bottom (at $\theta\approx+62^{o}$) or top (at $\theta\approx-62^{o}$) parts of the DNA strands closest to the positively charged row of amines (thick in Fig. S6b) are aligned along the row. In comparison with top parts, the bottom parts (thick black lines) are closer to the surface charges. For this reason, a deeper minimum is expected at $\theta\approx+62^{o}$. For the same reason (of unequal contributions from bottom and top phosphates), the central peak in Fig. S6b has an asymmetric shape. In these general features, the interaction of the helical DNA and non-chiral 1D periodical surface is expected to be similar to that of the pairwise interaction of two charged helices, where the additional energy minima different from the trivial one at $\vartheta=0$ appeared [S14]. As a consequence, more complex angular dependence of the transition from the linear to non-linear mechanical regimes is expected than shown in the phase diagram in Fig. 3d of the main paper. Noteworthy, $\pm62^{o}$ inclined arrangements corresponding to two side energy minima (Fig. S6b) qualitatively fit the proposed structural model of the confined irregularly undulating kinked DNA shown in Fig. 5j of the main paper.

Second, the overall consideration complexity is additionally increased by the interactions of the helical DNA with *several* charge rows it intersects (Figs. S6c and d). At the inclined $\boldsymbol{\theta\neq0}$ DNA arrangement, the electrostatic impact from the periodically charged surface follows "in phase" with the DNA helical structure only for several discrete inclinations $\boldsymbol{\theta_n}$ determined by the commensurability relationship $\mathbf{w/\sin\theta_n = np}$ (Fig. S6c), i.e. $\mathbf{\sin\theta_n = w/np}$,, where **w** is the surface period, **p** is the DNA helical pitch and **n** is integer. At *other* inclinations, $\boldsymbol{\theta\neq\theta_n}$, the incommensurability (δ) takes places (Fig. S6d) and, therefore, perturbations in the DNA structure can't be described by a periodic function.

The intrinsically large strength of the lateral electrostatic force inducing the overcritical bending and the incommensurability are two major factors making the DNA geometry both overcritically bent and frustrated and, therefore, principally anomalous. The theoretical consideration becomes dramatically complicated making the development of any analytical models of the DNA structural perturbations hardly possible.

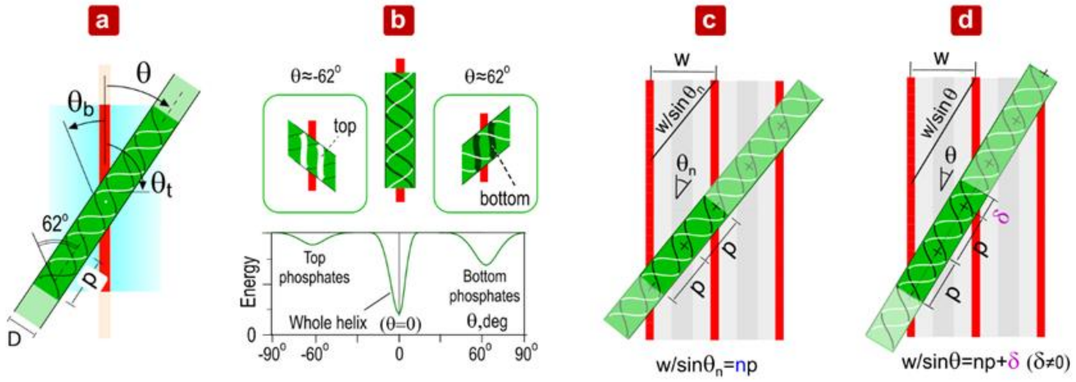

Fig. S6 The general features of the interaction of double-helical DNA with the one-dimensionally periodically charged surface. (**a**) The geometry of the DNA double helix interacting with a ***single*** row of surface charges. The bottom and top DNA phosphates are shown by black and white line segments respectively. (**b**) The general dependence of the interaction energy of the non-perturbed DNA segment on the interaxial angle θ. The depth and width of the central minimum at θ=0 are proportional and inversely proportional to the length of the DNA segment under the consideration, respectively. At the inclinations θ≈±62°, the major contribution is expected from the DNA phosphates closest and parallel to the row of positive surface charges (thick line segments in the sketches in the upper part of Fig. S6b), either bottom phosphates at θ≈62° or top phosphates at θ≈-62°. (**c, d**) At the DNA interaction with *several* rows of surface positive charges, the incommensurability of the DNA helical pitch ***p*** and the (inclination-dependent) periodicity of the lamellar electrostatic impact (w/sinθ) becomes principally important: (c) – the commensurable case, (d) – the incommensurable case (the electrostatic perturbations in the DNA structure can't be described by a periodic function).

Supplementary References

[S1] V.V. Prokhorov et al., High-Resolution Atomic Force Microscopy Study of Hexaglycylamide Epitaxial Structures on Graphite. Langmuir **27,** 5879–5890 (2011). https://doi.org/10.1021/la103051w

[S2] M.D. Frank-Kamenetskiĭ, V.V Anshelevich , A.V. Lukashin, Polyelectrolyte model of DNA. Sov. Phys. Usp. **30,** 317–330 (1987). https://doi.org/10.1070/PU1987v030n04ABEH002833

[S3] A.G. Cherstvy, Electrostatic interactions in biological DNA-related systems. Phys. Chem. Chem. Phys. **13,** 9942-9968 (2011). https://doi.org/10.1039/C0CP02796K

[S4] J. Lipfert, S. Doniach, R. Das, D. Herschlag, Understanding nucleic acid–ion interactions. Annu. Rev. Biochem. **83,** 813–841 (2014). https://doi.org/10.1146/annurev-biochem-060409-092720

[S5] G. Tellez, Nonlinear screening of charged macromolecules. Phil. Trans. Royal Soc. A **369,** 322–334 (2011). https://doi.org/10.1098/rsta.2010.0256

[S6] R. Cortini, X. Cheng, J. C. Smith, The tilt-dependent potential of mean force of a pair of DNA oligomers from all-atom molecular dynamics simulations. J. Phys. Condens. Matter **29**, 84002 (2017). https://doi.org/10.1088/1361-648X/aa4e68

[S7] C. Schildkraut, S. Lifson, Dependence of the melting temperature of DNA on salt concentration. Biopolymers **3**, 195–208 (1965). https://doi.org/10.1002/bip.360030207

[S8] S. Dasgupta, D.P. Allison, C.E. Snyder, S. Mitra, Base-unpaired Regions in Supercoiled Replicative Form DNA of Coliphage M13. J. Biol. Chem. **252**, 5916–5923 (1977).

[S9] D.I. Cherny, T.M. Jovin Electron and scanning force microscopy studies of alterations in supercoiled DNA tertiary structure. J. Mol. Biol. **313**, 295-307 (2001). https://doi.org/10.1006/jmbi.2001.5031

[S10] J. Adamcik, J.-H. Jeon, K.J. Karczewski, R. Metzler, G. Dietler, Quantifying supercoiling-induced denaturation bubbles in DNA. Soft Matter **8**, 8651 (2012). https://doi.org/10.1039/C2SM26089A

[S11] A.V. Vologodskii, A.V. Lukashin, V.V. Anshelevich, M.D. Frank-Kamenetskii, Fluctuations in superhelical DNA. Nucleic Acids Res. **6**, 967–982 (1979). https://doi.org/10.1093/nar/6.3.967

[S12] T.B. Liverpool, S.A. Harris, C.A. Laughton, Supercoiling and denaturation of DNA loops. Phys. Rev. Lett. **100**, 238103 (2008). https://doi.org/10.1103/PhysRevLett.100.238103

[S13] F. Sicard, N. Destainville, P. Rousseau, C. Tardin, M. Manghi, Dynamical control of denaturation bubble nucleation in supercoiled DNA minicircles. Phys. Rev. E **101**, 012403 (2020). https://doi.org/10.1103/PhysRevE.101.012403

[S14] A.A. Kornyshev, S. Leikin, Electrostatic interaction between long, rigid helical macromolecules at all interaxial angles. Phys. Rev. E **62**, 2576–2596 (2000). https://doi.org/10.1103/PhysRevE.62.2576
